# Increased CD56^bright^ NK cells in HIV-HCV co-infection and HCV mono-infection are associated with distinctive alterations of their phenotype

**DOI:** 10.1186/s12985-016-0507-5

**Published:** 2016-04-18

**Authors:** Suvercha Bhardwaj, Fareed Ahmad, Heiner Wedemeyer, Marcus Cornberg, Julian Schulze zur Wiesch, Jan van Lunzen, Shiv K. Sarin, Reinhold E. Schmidt, Dirk Meyer-Olson

**Affiliations:** Institute of Liver and Billiary Sciences, New Delhi, India; Klinik für Immunologie und Rheumatologie, Medizinische Hochschule Hannover, Hannover, Germany; Klinik für Gasteroenterologie, Hepatologie und Endokrinologie, Medizinische Hochschule Hannover, Hannover, Germany; Department of Medicine, University Medical Center, Hamburg-Eppendorf, Germany; Rheumatology and Internal Medicine, m & i–Fachklinik Bad Pyrmont, Bad Pyrmont, Germany; Viiv healthcare, London, UK; German Center for Infection Research (DZIF), Hamburg, Hannover Germany

## Abstract

**Background:**

HIV-HCV co-infection is associated with accelerated progression to hepatic fibrosis, cirrhosis and hepatocellular carcinoma than HCV mono-infection. The contribution of innate immunity during HIV-HCV co-infection has been a relatively under-investigated area. Natural killer (NK) cells are pivotal sentinels of innate immunity against viruses and tumour cells. In this study we evaluated the effect of HIV-HCV co-infection on peripheral blood NK cell subsets with emphasis on the phenotype of CD56^bright^ NK cells.

**Methods:**

Sixty patients were included in the study; HIV mono-infected (*n* = 12), HCV mono-infected (*n* = 15), HCV-HIV co-infected (*n* = 21) and healthy controls (*n* = 16). PBMCs were isolated and immunophenotyping of NK cells was performed by flowcytometry.

**Results:**

We observed an expansion of CD56^bright^ NK cell subset in HIV-HCV co-infection as compared to healthy controls and HIV mono-infected group. All the infected groups had an upregulated expression of the activating receptor NKG2D on CD56^bright^ NK cells in comparison to healthy controls while not differing amongst themselves.

The expression of NKp46 in HIV-HCV co-infected group was significantly upregulated as compared to both HIV as well as HCV mono-infections while NKp30 expression in the HIV-HCV co-infected group significantly differed as compared to HIV mono-infection. The CD56^bright^ NK cell subset was activated in HIV-HCV co-infection as assessed by the expression of CD69 as compared to healthy controls but was significantly downregulated in comparison to HIV mono-infection. CD95 expression on CD56^bright^ NK cells followed the same pattern where there was an increased expression of CD95 in HIV mono-infection and HIV-HCV co-infection as compared to healthy controls. In contrast to CD69 expression, CD95 expression in HCV mono-infection was decreased when compared to HIV mono-infection and HIV-HCV co-infection. Finally, expression of CXCR3 on CD56^bright ^NK cells was increased in HIV-HCV co-infection in comparison to HIV mono-infection while remaining similar to HCV mono-infection.

**Conclusion:**

Thus, HIV-HCV co-infection is able to modulate the phenotype of CD56^bright^ NK cell subset in a unique way such that NKp46 and CXCR3 expressions are distinct for co-infection while both mono-infections have an additive effect on CD56^bright^, CD69 with CD95 expressions. HCV mono-infection has a dominant effect on NKp30 expression while NKG2D and CD127 expressions remained same in all the groups.

## Background

NK cells are effector cells of the innate immune system, capable of destroying virus-infected and tumour cells without prior sensitization [1, 2]. The majority of human NK cells in peripheral blood are CD56^dim^CD16^+^ cells whereas CD56^bright^CD16^+/−^ cells only constitute approximately 10% of the peripheral blood NK cell pool [3]. In addition, CD56^bright^ NK cells have high surface density expression of type II membrane glycoprotein CD94, L-Selectin 62L, CD127 and lymph node homing receptor CCR7 but low expression of the low affinity IgG-Fc receptor III (CD16), killer cell immunoglobulin-like receptors (KIRs) and cytotoxic molecules such as perforin and granzyme B [4]. Thus, NK cell subsets seem to perform distinct roles in the immune response. CD56^bright^ NK cells have more regulatory functions by means of cytokine production while CD56^dim^ NK cells are primarily cytolytic in function but produce significant amounts of cytokines when their activating receptors are engaged [5]. This distinction is however not absolute [6].

The chemokine receptor repertoire also differ among the NK cell subsets with the expression of CXCR3 at a much stronger density on CD56^bright^ subset than CD56^dim^ cells, while the latter express CXCR1 and CX3CR1 exclusively [5, 7]. The differential repertoires of chemokine receptors and adhesion molecules endow NK cell subsets with divergent migratory properties: the CD56^bright^ subset preferentially homes to secondary lymphoid organs whereas the CD56^dim^ cells home to acute inflammatory sites [5, 7].

Persistent infections by HCV and HIV present unique challenges to the immune system as they result in highly chronic viral persistence which lasts indefinitely without antiviral treatment [8]. In HIV infection, several studies have shown functional impairment in NK cell cytokine secretion and cytotoxicity. Both acute and chronic untreated HIV infection is associated with alterations in NK cell subset distribution, with partial loss of CD56^+^CD16^+^ cells and expansion of CD56^−^CD16^+ ^NK cells having impaired cytolytic function, increased activation markers and decreased cytokine production [9, 10]. Similarly, HCV viral persistence has also been shown to be associated with defective NK cell responses in vivo both in the periphery and in the liver [7, 11]. NK cell function was rescued after successful IFN-α therapy in chronically HCV infected patients [12].

HIV infection is not only associated with changes in NK cell subpopulation but also with marked alterations in NK cell surface receptor expression and loss of function [13]. In HIV viremic patients, there is an overall decrease in surface receptor density of NKp46 and NKp30 found on freshly isolated NK cells and dysfunction in NKp44 *de novo* expression upon stimulation in vitro resulting in an NCR dull phenotype [14]. The proportion of NKp46 and NKp30 expressing CD56^dim^ NK cells and their cytolytic activity was shown to decrease with disease progression [15].

Contrary to HIV infection, there is no consensus on NCR expression during HCV infection. There are reports that show increased proportions and density of NCRs including NKG2C, NKp44, NKp30 and NKp46 [16, 17] while the earlier reports of decreased expression of NKp46 have not been subsequently confirmed [7]. In addition, couple of recent studies have suggested that HCV infected cells may selectively down regulate NKp30 and impair NK cell function by this mechanism [14, 18, 19].

Nearly all NK cells express NKG2D which is considered a potent activating receptor [20] that has the ability to trigger cytotoxicity and at the same time capable of overriding signals provided by other inhibitory receptors. Similar to other NCRs, there is conflicting evidence with respect to NKG2D expression which has been reported from being up-regulated or down-regulated to being unchanged during chronic HCV infection [16, 21].

A number of studies have revealed CD56^bright^ and CD56^dim^ NK cells as separate NK cell subsets rather than a homogenous population having unique roles in the innate immune response [7]. By virtue of their ability to produce different cytokines, CD56^bright^ NK cells might play an important role in early immune responses as well as in shaping of the adaptive response [5]. Not much is known about the impact of HIV and HCV co-infection on CD56^bright^ NK cells. In the current study, we therefore investigated the phenotype of CD56^bright^ NK cells in HIV-HCV co-infected subjects and compared these with HCV and HIV mono-infected patients as well as with healthy controls. We found that HIV-HCV co-infection is able to modulate the phenotype of CD56^bright^ NK cells in a complex way.

## Results

### Frequency of CD56^bright^ NK cells

**Fig. 1:**
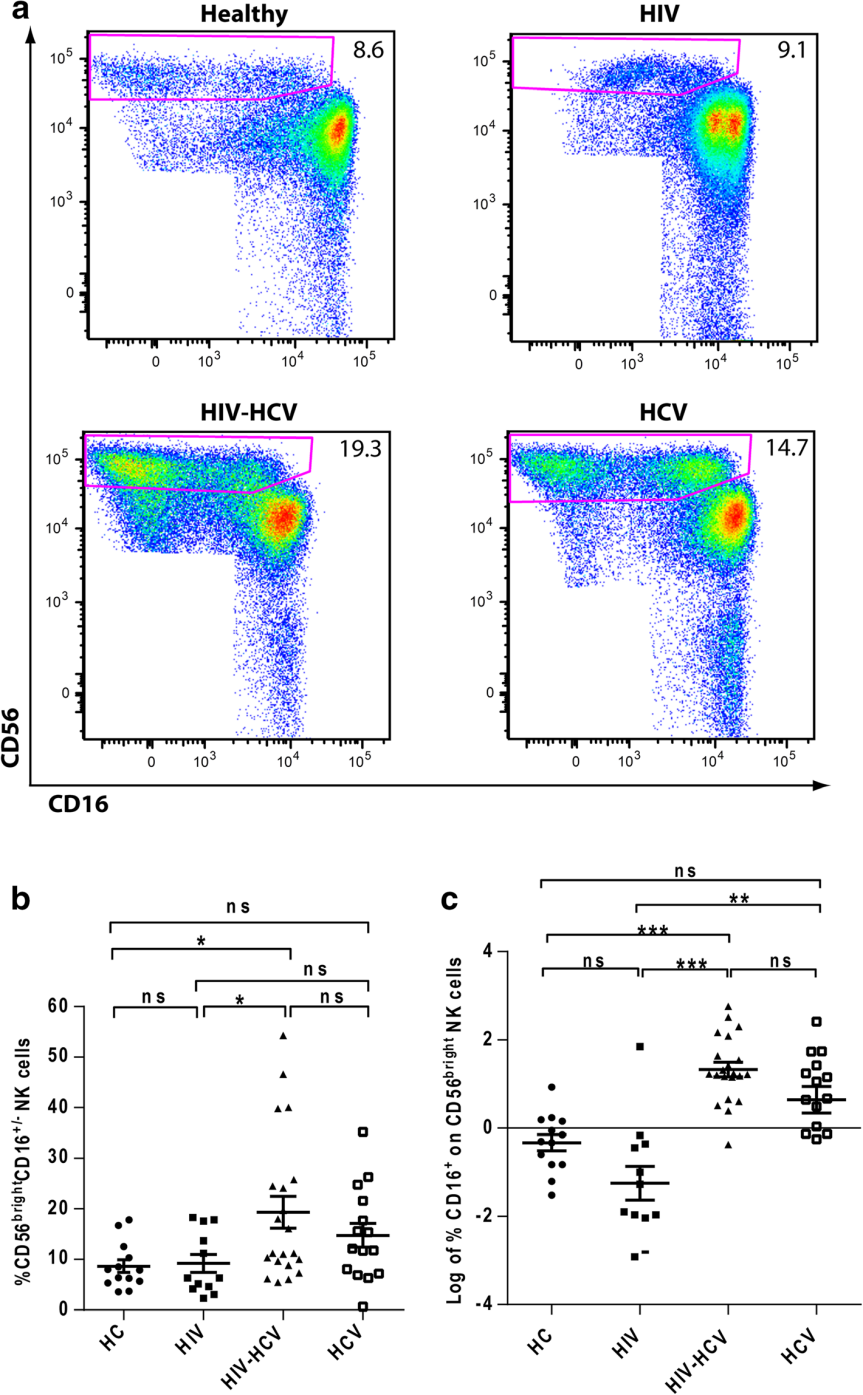
Increased CD56^bright^CD16^+/-^ NK cells in HIV-HCV co-infection. **a** Representative gating scheme for identification of CD56^bright^CD16^+/-^ NK cells. **b** Comparison of percentage of the CD56^bright^ NK cell subpopulation in control subjects (*n* = 13), HIV seropositive (*n* = 12), HIV-HCV co-infected (*n* = 21) and HCV mono-infected individuals (*n* = 15) on CD56^bright^ CD16^+/−^ NK cells. (**c**) Comparison of log percentage of the CD56^bright^ NK cells expressing CD16 in control subjects (n = 13), HIV seropositive (n = 12), HIV-HCV co-infected (n = 21) and HCV mono-infected individuals (n = 15) on CD56^+^CD16^+/-^ NK cells. *, *P* < 0.05; *ns -* not significant (*P* > 0.05). In the dot plot figure horizontal line represents % Mean ± SEM and Log % Mean ± SEM respectively

We defined NK cells as CD3^−^CD14^−^CD19^−^ lymphocytes expressing either CD16 or CD56 or both as described previously [10]. Utilizing CD56 and CD16 we defined CD56^bright^ NK cell population in peripheral blood as shown in Fig. 1a. The percentage of CD56^bright^CD16^+/−^ NK cells in HIV and HCV mono-infections did not differ significantly as compared to the healthy controls. On the other hand HIV-HCV co-infection had significant upregulation of CD56^bright^CD16^+/−^ NK cells as compared to healthy controls. As compared to mono-infections HIV-HCV co-infection had an upregulated expression of CD56^bright^CD16^+/− ^NK cells than HIV mono-infection only. Although HCV mono-infection showed a trend towards increased CD56^bright^CD16^+/− ^NK cells, only HIV-HCV co-infection resulted in an increase that was significantly different from both healthy controls and HIV mono-infection (Fig. 1b). The log percentage of CD56^bright^ NK cells expressing CD16 in HIV-HCV co-infected patients also differed significantly as compared to healthy controls and HIV mono-infected group (Fig. 1c).

Our data indicate that HIV-HCV co-infection is associated with an increase of CD56^bright ^NK cells. In addition CD16^+^CD56^bright^ NK cells in all the infected groups had a significant positive correlation with CD56^bright^CD16^+/−^ NK cells (Table 1).

**Table 1:** Correlation between various NK cell receptors

HIV mono-infection		CD56^bright^CD16^+/−^ NK cells	NKp30^+^CD56^bright ^NK cells
CD16^+^CD56^bright^ NK cells	Correlation coefficient	r_s_ = 0.742**	NA
	Sig. (2 tailed)	*P* < 0.01	NA
NKp46^+^CD56^bright^ NK cells	Correlation coefficient	NA	r_s_ = 0.633*
	Sig. (2 tailed)	NA	*P* < 0.05
HIV-HCV co-infection		CD56^bright^CD16^+/− ^NK cells	CD127^+^CD56^bright^ NK cells
CD16^+^CD56^bright^ NK cells	Correlation coefficient	r_s_ = 0.810**	NA
	Sig. (2 tailed)	*P* < 0.01	NA
CD69^+^CD56^bright^ NK cells	Correlation coefficient	NA	r_s_ = −0.433*
	Sig. (2 tailed)	NA	*P* < 0.05
HCV mono-infection		CXCR3^+^CD56^bright ^NK cells	
NKp46^+^CD56^bright^ NK cells	Correlation coefficient	r_s_ = 0.587*	NA
	Sig. (2 tailed)	*P* < 0.05	NA

*r*_*s*_ Spearman coefficient; *NA* not applicable; *, *P* < 0.05; **, *P* < 0.01

### Expression of NKG2D and NCRs

**Fig. 2:**
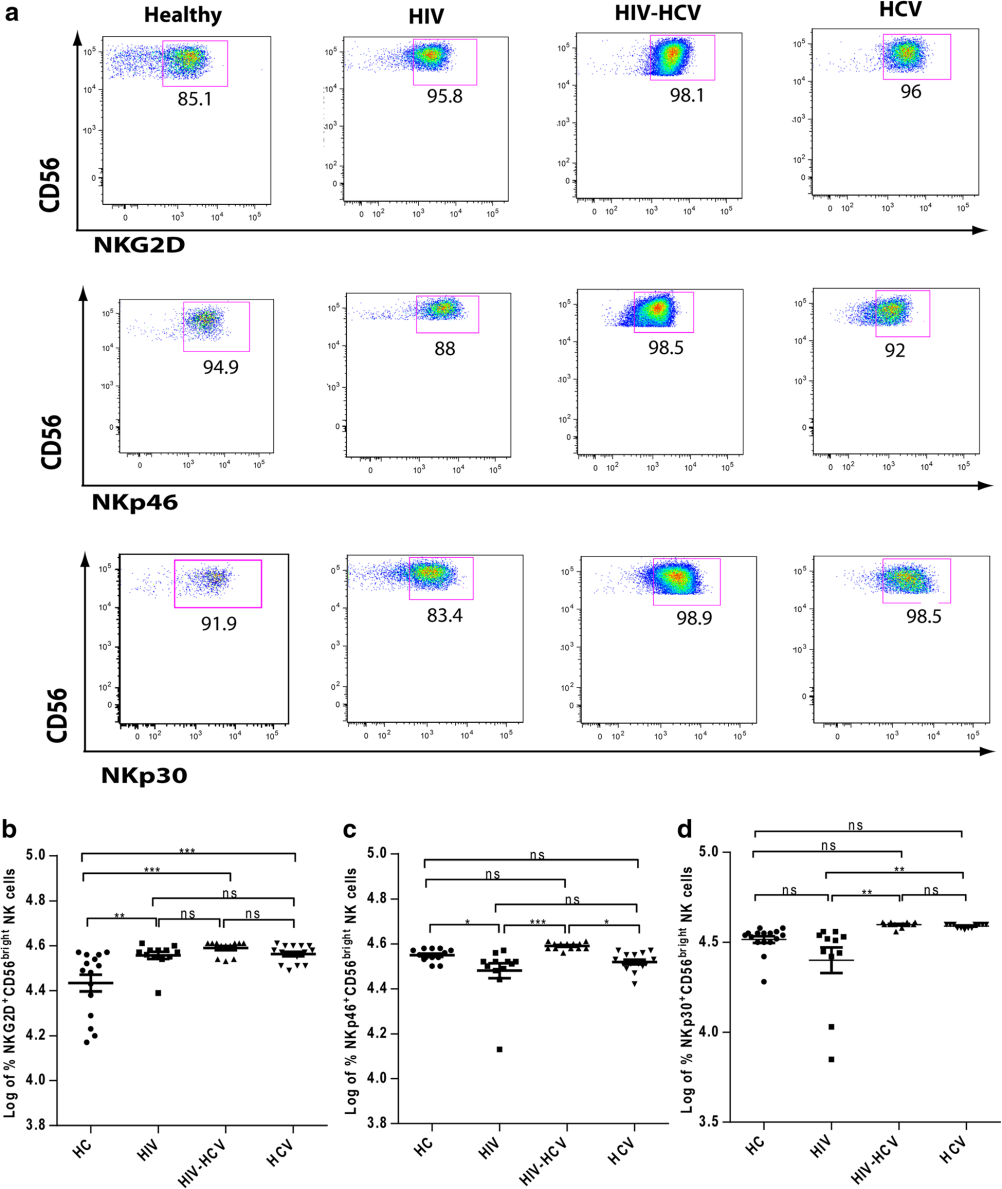
Upregulated expression of NKG2D and NCRs on CD56^bright^CD16^+/-^ NK cells in HIV-HCV co-infection. **a** Representative examples of flow cytometric analysis of C-type lectin receptor NKG2D and NCRs NKp46 and NKp30 expression on CD56^bright^CD16^+/-^ NK cells. **b** Surface expression of NKG2D on CD56^bright^CD16^+/-^ NK cell subset in healthy controls (*n* = 15), HIV mono-infected (*n* =12), HIV-HCV co-infected (*n* = 12) and HCV mono-infected patients (*n* = 15). **c** Surface expression of NKp46 on CD56^bright^CD16^+/-^ NK cell subset in healthy controls (*n* = 13), HIV mono-infected (*n* =11), HIV-HCV co-infected (*n* = 13) and HCV mono-infected patients (*n* = 15). **d** Surface expression of NKp30 on CD56^bright^CD16^+/-^ NK cell subset in healthy controls (*n* = 16), HIV mono-infected (*n* =11), HIV-HCV co-infected (*n* = 12) and HCV mono-infected patients (*n* = 12). *, *P* < 0.05; **, *P* < 0.01; ***, *P* < 0.001; *ns -* not significant (*P* > 0.05). In the dot plot figures horizontal line represents Log % Mean ± SEM

The representative flow cytometry plots of NKG2D and NCRs, NKp46 and NKp30 expression on CD56^bright^ NK cells is shown in Fig. 2a. All the infected groups did not differ significantly for NKG2D expression on the CD56^bright^ NK cell subset; however its expression was significantly up-regulated on all the infected groups as compared to healthy controls (Fig. 2b).

The expression of NKp46 on CD56^bright^ NK cells was significantly down-regulated in HIV mono-infected group as compared to healthy controls (Fig. 2c). In contrast, it was up-regulated on HIV-HCV co-infected group as compared to HIV and HCV mono-infected groups. However, the frequency of NKp46^+^CD56^bright ^NK cells did not differ significantly between HIV and HCV mono-infections (Fig. 2c).

The log percentage of NKp30^+^CD56^bright^ NK cells in all the infected groups did not differ significantly as compared to healthy controls. NKp30 expression on CD56^bright^ NK cells was significantly downregulated in HIV mono-infected group than both HIV-HCV co-infection and HCV mono-infection (Fig. 2d) while in case of HCV mono-infected group NKp30 expression differed significantly as compared to HIV mono-infected group only (Fig. 2d). Also in HIV mono-infected group the expression of NKp46 on CD56^bright^ NK cells had a direct correlation with the expression of NKp30 on CD56^bright ^NK cells (Table 1), while in HCV mono-infected patients NKp46^+^CD56^bright^ NK cells were positively correlated with the expression of CXCR3 on CD56^bright ^NK cells (Table 1).

### Activation status, expression of Fas receptor (CD95) and proliferative capacity of CD56^bright^ NK cells

**Fig. 3:**
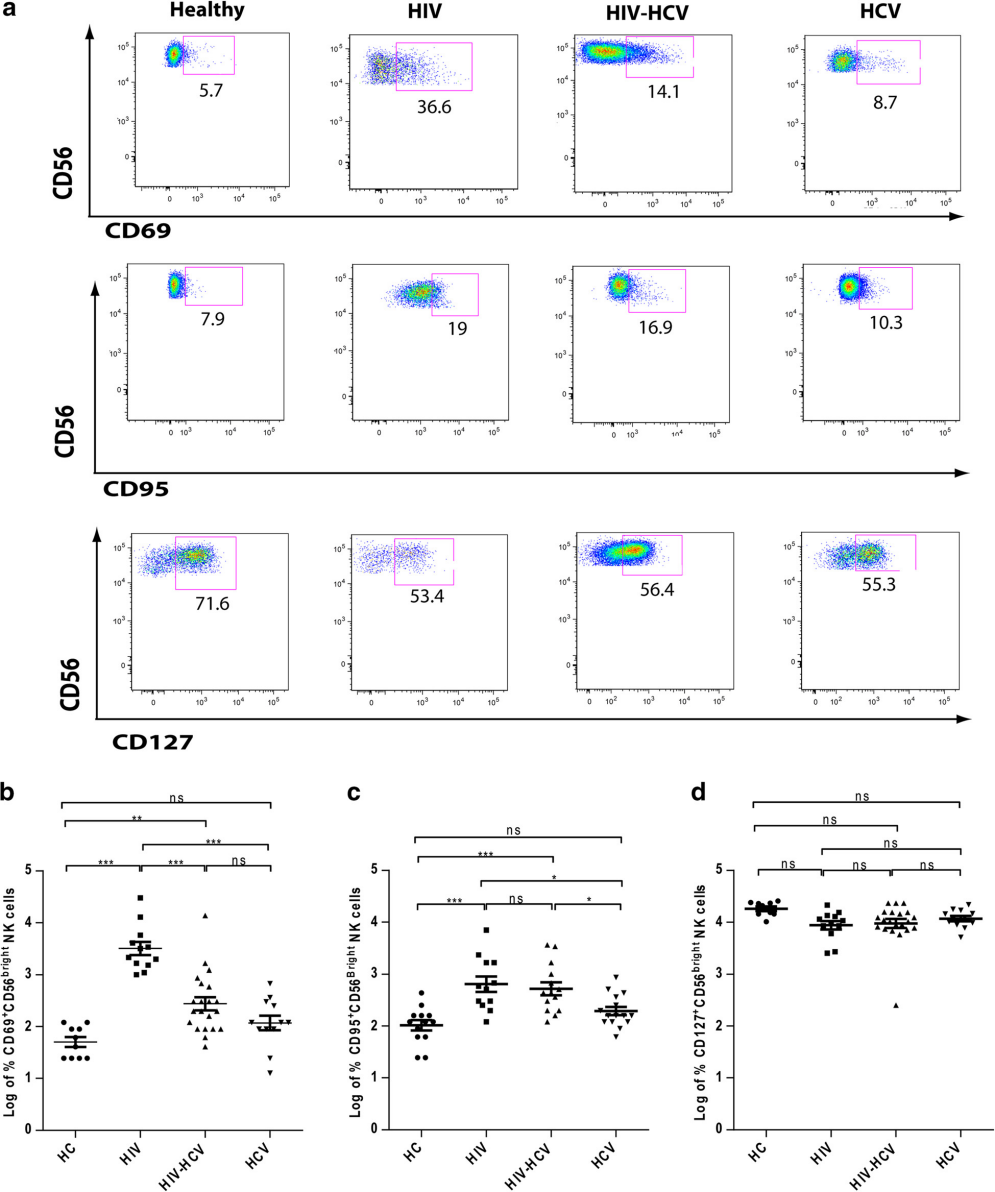
Expression of CD69, CD95 and CD127. **a** Representative dot plots obtained from the measurement of CD69, CD95 and CD127 expression on CD56^bright^CD16^+/-^ NK cells by flow cytometry. **b** CD69 expression on CD56^bright^CD16^+/-^ NK cells in healthy controls (*n* = 10), HIV mono-infected (*n* =12), HIV-HCV co-infected (*n* = 21) and HCV mono-infected patients (*n* = 12). **c** CD95 expression on CD56^bright^CD16^+/- ^NK cells in healthy controls (*n* = 13), HIV mono-infected (*n* =12), HIV-HCV co-infected (*n* = 14) and HCV mono-infected patients (*n* = 15). **d** CD127 expression on CD56^bright^CD16^+/-^ NK cells in healthy controls (*n* = 10), HIV mono-infected (*n* =12), HIV-HCV co-infected (*n* = 21) and HCV mono-infected patients (*n* = 12). *, *P* < 0.05; **, *P* < 0.01; ***, *P* < 0.001; *ns -* not significant (*P* > 0.05). In the dot plot figures horizontal line represents % Mean ± SEM

Figure 3a shows the representative flow cytometry plots of CD69, CD95 and CD127 expressions on CD56^bright^ NK cells. The log percentage expression of CD69 which is an early activation marker [22], was found to be considerably increased among CD56^bright^ NK cells in HIV mono-infected and HIV-HCV co-infected groups in comparison to healthy controls suggesting a heightened status of immune activation in HIV mono-infected and HIV-HCV co-infected patients (Fig. 3b). Among the infected groups HIV-HCV co-infected and HCV mono-infected groups showed lower CD69 expression than HIV mono-infected group (Fig. 3b). The finding of a more ‘HCV-like’ phenotype in HIV-HCV co-infection suggests that reduced CD69 expression might also be caused by an increase of CD56^bright^ NK cells that lack CD69 in the HIV-HCV co-infected cohort.

During chronic viral infections, activated immune cells (lymphocytes) or the highly exhausted cells are deleted by apoptosis [23]. CD95 (Fas receptor) binds to CD95L (Fas ligand) and induces apoptosis. The expression of CD95 on CD56^bright^ NK cells was upregulated in both HIV mono-infected and HIV-HCV co-infected groups as compared to healthy controls (Fig. 3c). At the same time, CD95 expression was downregulated on the CD56^bright^ NK cell subset in HCV mono-infection in comparison to both HIV mono-infection and HIV-HCV co-infection (Fig. 3c).

The expression of CD127 on CD56^bright^ NK cells is associated with high proliferative capacity. Expression of CD127 was downregulated in all the infected groups as compared to healthy controls, although this decrease was not significant (Fig. 3d). Between the infected groups there was no difference for CD127 expression on CD56^bright^ NK cells (Fig. 3d).

In addition, the expression of CD69 on CD56^bright^NK cells had a negative correlation with CD127^+^CD56^bright^ NK cells (Table 1).

### Chemokine receptor CXCR3 expression

**Fig. 4:**
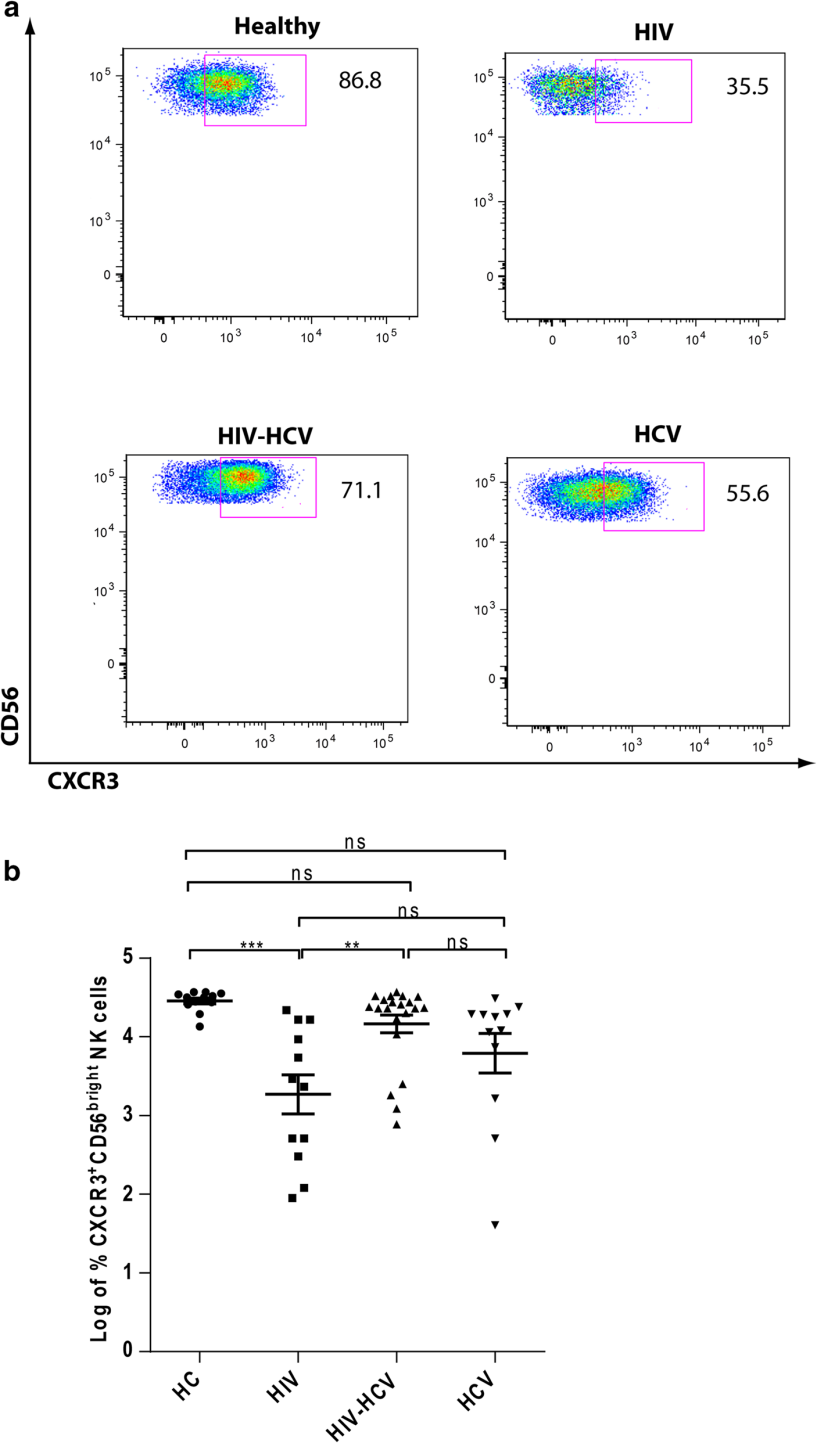
Increased chemokine receptor CXCR3 expression on CD56^bright^CD16^+/−^ NK cells in HIV-HCV co-infection. **a** Representative dot plots obtained from the measurement of CXCR3 expression on CD56^bright^CD16^+/−^ NK cells by flow cytometry. **b** CXCR3 expression on CD56^bright^CD16^+/−^ NK cells in healthy controls (*n* = 10), HIV mono-infected (*n* =12), HIV-HCV co-infected (*n* = 21) and HCV mono-infected patients (*n* = 12). **, *P* < 0.01; ***, *P* < 0.001; *ns -* not significant (*P* > 0.05). In the dot plot figure horizontal line represents Log % Mean ± SEM

Among the NK cell subsets CXCR3 is expressed more on CD56^bright^ than CD56^dim^ subset. As compared to healthy controls, the expression of CXCR3 on CD56^bright^ NK cells was significantly down-regulated in HIV mono-infected group (Fig. 4b). Interestingly, CXCR3 expression was higher on CD56^bright^ NK cells in the HIV-HCV co-infected group as compared to the HIV mono-infected group (Fig. 4b).

Finally we did not find any correlation between the HCV or HIV viral load with the fractions of different CD56^bright^ NK subsets that were assessed.

## Discussion

HIV-HCV co-infected individuals progress more rapidly to fibrosis, cirrhosis, liver failure and hepatocellular carcinoma than patients infected with HCV alone [7, 24]. Both HIV and HCV mono-infections have been shown to have impaired NK cell functions and skewed subset distributions [7, 9]. However, the impact of HIV-HCV co-infection on phenotypic alterations of NK cell subsets is not well understood. Here, we investigated the phenotype of CD56^bright^ NK cells in HIV-HCV co-infected individuals and compared it with HIV or HCV mono infected patients as well as with healthy controls. In our study we found an expansion of CD56^bright^ NK cell subset in HIV-HCV co-infected cohort similar to reported for HCV mono-infection [25, 26] although there is a study reporting no change in CD56^bright^ NK cell compartment in HCV mono-infection [27]. In line with our findings, in a cohort of female patients chronically infected with HCV, the proportion of CD56^bright^ NK cells was increased as compared to HCV resolvers and healthy controls [25]. The same increased percentage of CD56^bright ^NK cells was found among individuals with a positive tuberculin skin test compared with patients with overt tuberculosis and normal controls [28]. The significance of increased CD56^bright ^ cells in our study is currently unclear. Expansion of CD56^bright^ NK cells in HCV infection might have resulted from a decreased rate of differentiation towards CD56^dim^ NK cells in these studies. However, in our study the percentage of total NK cells and CD56^dim^ NK cells did not differ among all the groups (data not shown). The increased percentage of CD16 expression on CD56^bright ^NK cells in HIV-HCV co-infected patients indicated a phenotypic shift towards CD56^dim^ NK cells and their subsequent role in antibody dependent cellular cytotoxicity (ADCC). Increased expression of CD16 on CD56^bright^ NK cells is also reported in HIV-1 infected individuals which is partially restored after ART [29].

NK cell function is primarily regulated by balance between activating and inhibitory receptors. NKG2D and NCRs are one of the main NK cell activating receptors that are required for NK cell effector functions. NKG2D and NCRs are differentially expressed by different subsets of NK cells [20, 30]. We analysed the expression of NKG2D and NCRs (NKp30 and NKp46) on CD56^bright^ NK cell subset. NKG2D was upregulated in all the three infected populations as compared to healthy controls. The cytotoxic potential of all NK cell subsets increases when stimulated with cytokines like IL-2 or IL-12 in vitro [7]. This might be one of the reasons for the increased expression of NCRs and NKG2D on CD56^bright^ NK cells in vivo as well in chronic viral infections.

In HIV viremic patients, there is an overall decrease of NKp46 and NKp30 on NK cells [14] while in HCV infection couple of studies have shown reduced NKp30^+^ NK cells. In our study, we did not observe a decreased NKp30 expression on CD56^bright^ NK cells in HCV mono-infection. Thus, reduced NKp46 and NKp30 on CD56^bright ^NK cells is a unique hallmark of HIV infection [14, 18, 19]. However these results are not directly comparable or necessarily in conflict as our results are focussed on the different fractions of CD56^bright^ NK cell subset.

In our study NKp46 expression on HIV-HCV co-infected group differed significantly from both HIV and HCV mono-infected groups while NKp30 expression was increased on CD56^bright^ NK cell subset in HIV-HCV co-infected cohort which differed significantly as compared to HIV mono-infected group. The finding of increased frequency of NKp30 and NKp46 expressing CD56^bright^ NK cells in HIV-HCV co-infection as compared to HIV mono-infection also supports a shift of the populations either due to increased apoptosis of activated CD56^bright^ NK cells or an increase of ‘HCV-like’ CD56^bright^ NK cells in case of NKp30 expression while indicating a unique and an important role of NKp46 in HIV-HCV co-infection.

Immune activation is a hallmark of chronic viral infections like HIV [31]. HIV infected patients showed an increased activation of CD56^bright^ NK cells as compared to HIV-HCV co-infected and HCV mono-infected groups as measured by the expression of CD69. This might be because of the ongoing residual viral replication even after one year of ART [32]. The reduced expression of CD69 in HIV-HCV co-infected cohort suggests a decreased activation status of CD56^bright^ NK cells in HIV-HCV co-infection than HIV mono-infection. However, it is unlikely that HCV co-infection might ‘protect’ CD56^bright^ NK cells from increased immune activation. Alternatively, a loss of CD69 expressing CD56^bright^ NK cells could be either due to increased apoptosis or a relative increase of CD69 negative NK cells in the HIV-HCV co-infected cohort or both. We therefore analysed the expression of the apoptosis marker CD95.

Immune cells undergo apoptosis as a result of chronic activation or exhaustion during chronic viral infections. Fas mediated apoptosis (FMA) is one of the mechanisms by which cells undergo apoptosis [33]. The expression of Fas receptor CD95 on CD56^bright^ NK subset was higher in HIV mono-infected patients. The same group also had the high expression of CD69 on the CD56^bright^ NK cells showing a chronic systemic immune activation status. The increased expression of CD69 as well as CD95 on CD56^bright^ NK cell subset in HIV treated patients may render activated NK cells more prone to undergo apoptosis. Enhanced susceptibility of NK cells to cell death by CD95-CD95L in HIV infected viremic individuals has already been reported by Kottilil *et al*. [34]. A number of reports show increased expression of CD95 on various lymphocyte subsets as a result of immune activation induced by HIV viremia thereby facilitating lymphocyte apoptosis [35, 36]. The expression of CD69 on CD56^bright^ NK cells in HIV-HCV co-infected group was closer to HCV mono-infected group whereas the CD95 expression behaved more similar to HIV treated mono-infected group thus showing imprints of either infection.

CD127 (IL-7Rα receptor) is important for homeostatic proliferation, particularly in lymphopenic settings when remaining cells initiate a homeostatic response to repopulate the depleted compartment. The level of IL-7 and expression patterns of CD127 are believed to be skewed in HIV [37, 38]. Our study also demonstrated a skewed expression of CD127 on CD56^bright^ NK cells in all the infected groups as compared to healthy controls, but the difference was not significant. Chronic viral infections may be associated with reduced CD127 in general.

Chemokine receptor CXCR3 and its ligands (particularly IP-10) are associated with a type 1 response and recruit lymphocytes to the liver [39, 40]. Kimball *et al.* [41] have previously demonstrated an increased CXCR3 expression on CD8^+^T cells from HIV-HCV co-infected patients as compared to healthy controls. A recent study showed HCV infection to be associated with significantly increased frequency of CXCR3^+^ CD56^bright^ NK cells but these cells showed an impaired degranulation and IFN-γ secretion in response to hepatic stellate cells (HSCs) [42]. In our study, we observed a decreased expression of the frequency of CXCR3^+^ CD56^bright^ NK cells in HCV mono-infection as well as in HIV mono-infection, which was even more pronounced than in HCV mono-infection. In addition, our study showed an elevated expression of CXCR3 on CD56^bright^ NK cells in HIV-HCV co-infection as compared to HIV and HCV mono-infected groups which may reflect an effort to recruit more CD56^bright^ NK cells to liver in the wake of a co-infection.

One of the limitations of the current study is the lack of NK cell functional data in terms of NK cell cytotoxicity and cytokine secretion. Functional assays will reflect more clearly on the real impact of HIV or HCV mono-infection on HIV-HCV co-infection and may also help in deciphering the complex phenotype observed in HIV-HCV co-infection in our study.

In summary, the increased CD56^bright^ NK cells in HIV-HCV co-infection might be a compensatory mechanism to recruit more immunoregulatory CD56^bright^ NK cells. Even though NKp30 and NKp40 expression was increased in HIV-HCV co-infection, NKp40 expression was unique for HIV-HCV co-infection as it behaved neither like HIV nor HCV mono-infection. This observation may imply that among NCRs, NKp46 seems to play an important role in the cytotoxic potential of CD56^bright^ NK cells and in combating both HIV and HCV mono-infections. NKp30 expression was same for HCV mono-infection and HIV-HCV co-infection indicating that chronic viral hepatic infections do not have a dominant bearing on the expression of NKp30 on CD56^bright^ NK cells. While HIV and HCV mono-infections differed for NKp30 expression, they remained same for NKp46 expression on CD56^bright^ NK cells. Increased NKG2D expression in HIV and HCV mono-infections and HIV-HCV co-infection rather reflects a general feature of viral infections. Interestingly, CXCR3 expression increased in HIV-HCV co-infection vis-à-vis in HIV and HCV mono-infections - probably caused by an additional infection. It can also be speculated that increased CD69 and CD95 expression on CD56^bright^ NK cells in HIV mono-infection are a reflection of their exhaustive phenotype. A summary of various NK cell receptors expression is provided in Table 2.

**Table 2:** Summary of various NK cell receptors expression data

NK cell receptor	HIV vs HC	HIV-HCV vs HC	HCV vs HC	HIV-HCV vs HIV	HIV-HCV vs HCV	HIV vs HCV
CD56^bright^ NK cells	−	↑	−	↑	−	−
CD16^+^CD56^bright^ NK cells	−	↑↑↑	−	↑↑↑	↑↑	−
NKG2D^+^CD56^bright^ NK cells	↑↑	↑↑↑	↑↑↑	−	−	−
NKp46^+^CD56^bright^ NK cells	↓	−	−	↑↑↑	↑	−
NKp30^+^CD56^bright^ NK cells	−	−	−	↑↑	−	↓↓
CD69^+^CD56^bright^ NK cells	↑↑↑	↑↑	−	↓↓↓	−	↑↑↑
CD95^+^CD56^bright ^NK cells	↑↑↑	↑↑↑	−	−	↑	↑
CD127^+^CD56^bright^ NK cells	−	−	−	−	−	−
CXCR3^+^CD56^bright^ NK cells	↑↑↑	−	−	↑↑	−	−

Direction of the arrows documents upregulation or downregulation of NK receptor expression in respective comparisons. ↑ or ↓ = *P* < 0.05; ↑↑ or ↓↓ = *P* < 0.01; ↑↑↑ or ↓↓↓ = *P* < 0.001

## Conclusion

HIV-HCV co-infection exhibits a complex imprinting of the CD56^bright^ NK cell phenotype. HIV-HCV co-infection is characterized by an increased CD56^bright^ NK cell population. NKG2D expression behaves similarily in all the infected groups. NKp46 expression is higher in HIV-HCV co-infection than in both mono-infections and is thus distinct for HIV-HCV co-infection, while NKp30 expression behaves more like in HCV mono-infection. The expression of the activation marker CD69 and FasR CD95 is in between HIV and HCV mono-infections (closer to HCV mono-infection and closer to HIV mono-infection respectively) but clearly influenced by both HIV and HCV mono-infections for CD69 and CD95 expressions. CD127 expression is same in all the groups. Expression of CXCR3 in co-infection is clearly different as it is not reduced as much as in either mono-infections so this combination is also unique for HIV-HCV co-infection. Thus we see a complex phenotype of CD56^bright^ NK cells in HIV-HCV co-infection. Future functional studies may investigate as to what extend these altered phenotypes may influence the course of co-infection and which factors are responsible for each individual to define how HIV-HCV co-infection affects the CD56^bright^ NK cells.

## Methods

### Study subjects

We obtained peripheral blood samples from 12 HIV seropositive subjects both treated [11] and untreated [1], 15 HCV mono-infected untreated subjects with replicative HCV infection, 21 HIV-HCV co-infected subjects both treated [17] and untreated [4] for HIV in the HIV outpatient clinic of the Medizinische Hochschule Hannover (MHH) but treatment naïve for HCV and 16 healthy controls. All HCV infected subjects were also treatment naive for hepatitis C during the study period. All study subjects were recruited at the HIV and HCV outpatient clinic of the Medizinische Hochschule Hannover (MHH) and of the University Medical Centre, Hamburg-Eppendorf. All study participants gave written, informed consent prior to their participation. The study was approved by the local ethics committee (Votum der Ethikkommission der MHH No. 3150).

A summary of the demographical data of the studied groups is shown in Table 3. Plasma HIV-1 RNA level was measured with the COBAS TaqMan HIV-1 test (Roche Diagnostics, Basel, Switzerland) with a lower limit of detection of 34 copies/ml.

**Table 3:** Summary and comparison of log values of demographic characteristics of study groups

Parameters	HC	HIV	HIV-HCV	HCV	*P* value
	(*n*=16)	(*n*=12)	(*n*=21)	(*n*=15)	
Mean age (years)±SD	43.8±3.3	45±5.5	47±7.6	48.5±5.8	0.15
Male/Female	7/5	8/4	16/5	7/8	0.34
HIV RNA, mean copies/ml	Seronegative	6.1±3.5	5.7±3.0	N/A	0.76
Mean CD4^+^ T cell count (n/µl)±SD	NT	6.1±0.5	5.8±0.7	NT	0.24
Treated/Untreated for HIV	N/A	11/1	17/4	N/A	0.41
HCV RNA, mean copies/ml	Seronegative	N/A	13.0±2.9	14.5±1.1	0.12
				*n*=4 (NA)	
AST (U/L), mean			4.2±0.5	3.9±0.5	0.1
	NT	N/A	*n*=2 (NA)	*n*=1 (NA)	
ALT (U/L), mean			4.2±0.7	4.0±0.7	0.36
	NT	N/A	*n*=2 (NA)	*n*=1 (NA)	
γGT (U/L), mean			4.4±0.8	4.0±0.8	0.15
	NT	N/A	*n*=2 (NA)	*n*=1 (NA)	
Billirubin (µmol/L), mean			2.3±0.6	2.4±0.4	0.72
	NT	N/A	*n*=2 (NA)	*n*=1 (NA)	

*Abbreviations*: *N/A* not applicable, *NT* not tested, *NA* not available

Absolute counts of CD4^+^ T cells, NK cells and other lymphocyte subpopulations were determined by flow cytometry using a cocktail of antibodies from Beckman Coulter, California, USA either directed against CD45, CD3, CD4 and CD8 or CD45, CD56, CD19, CD3 and CD16.

### Isolation of mononuclear cells

PBMCs were isolated from fresh blood as described previously [17]. Aliquots of 10^7^ PBMCs each were cryopreserved in heat-inactivated FCS supplemented with 10 % dimethyl sulfoxide (DMSO) (Merck, NJ, USA).

### Phenotypic analysis of NK cells by flowcytometry

Staining and flow cytometric analysis was performed as described before [10]. The Following monoclonal antibodies were used in this study: Via probe PerCP, anti-CD19 PerCP, anti-CD14 PerCP and anti-CD3 PerCP (BD Biosciences, CA, USA) to exclude dead cells, B cells, monocytes and T cells, respectively, and anti-CD56 PC7 (Beckman Coulter, CA, USA) and anti-CD16 APC-H7 (BD Biosciences, CA, USA) to identify NK cells. Additional antibodies that were used include: anti-CXCR3 APC (BD Biosciences, CA, USA), anti-CD69 PE (Invitrogen, CA, USA), anti-CD127 PacBlue (BD Biosciences, CA, USA), anti-CD95 APC (Biolegend, CA, USA), anti-NKp30 APC (Beckman Coulter, CA, USA), anti-NKp46 PE (Beckman Coulter, CA, USA), anti-NKG2D APC (BD Biosciences, CA, USA). At least 1 million events were acquired for each sample, using the BD Canto II (BD Biosciences, CA, USA). Data were analysed with FlowJo 8.8.4 (TreeStar, Or, USA). Lymphocytes were defined by forward and side scatter. CD3^+^, CD14^+^, CD19^+^, dead cells and cell aggregates were removed from analysis based on PerCP and Viaprobe cell viability staining and pulse width analysis. Fluorescence minus one (FMO) staining was used to determine threshold values for the expression of each surface marker.

### Statistical analysis

GraphPad Prism (version 5.0) software was used for statistical evaluation of the data. One-way ANOVA followed by post hoc comparison by Bonferroni method was performed. Kruskal-Wallis test was applied for CD16^+^CD56^bright^ NK cells followed by multiple comparisons by Mann-Whitney after adjusting the probability level. The correlation between various parameters was studied using Pearson/Spearman correlation. The categorical data was analysed by chi-square/Fisher’s exact test wherever necessary. The log transformation was also applied for making the skewed data normalized. *P* values less than 0.05 were considered significant.
